# Development of an immunochromatographic strip for the serodiagnosis of *Theileria* infection in sheep

**DOI:** 10.1186/s13071-015-1234-2

**Published:** 2015-12-02

**Authors:** Yizhu Lu, Guiquan Guan, Tao Jiang, Youquan Li, Jifei Yang, Guangyuan Liu, Jianxun Luo, Hong Yin, Zhijie Liu

**Affiliations:** State Key Laboratory of Veterinary Etiological Biology, Key Laboratory of Veterinary Parasitology of Gansu Province, Lanzhou Veterinary Research Institute, Chinese Academy of Agricultural Sciences, Xujiaping 1, Lanzhou, Gansu 730046 P. R. China; Jiangsu Co-innovation Center for Prevention and Control of Important Animal Infectious Diseases, Yangzhou, China

**Keywords:** *Theileria uilenbergi*, *Theileria luwenshuni*, Colloidal gold, Immunochromatographic strip test

## Abstract

**Background:**

*Theileria uilenbergi* and *T. luwenshuni* are tick-borne protozoan parasites, transmitted by *Haemaphysalis qinghaiensis* and *H. longicornis*, respectively. They are the main causative agents of theileriosis in small ruminants in China. The disease has resulted in severe economic losses and hindered the development of sheep and goat husbandry industry in the endemic regions.

**Methods:**

In this study, a colloidal gold-based immunochromatographic strip (ICS) was developed for the detection of *T. uilenbergi* and/or *T. luwenshuni* infections. A recombinant *T. uilenbergi* immunodominant protein (rTuIP) was used as antigen for the ICS. The nitrocellulose membrane was incubated with rTuIP on the test (T) line and anti-rTuIP antiserum on the control (C) line, respectively. The rTuIP conjugated to colloidal gold particles was used as the detection system for visualization of the lines. Then the sample pad, conjugate pad, nitrocellulose membrane and absorbent pad were assembled onto a backing plate in the appropriate order.

**Results:**

The ICS was able to detect antibodies in the sera of animals experimentally infected with *T. uilenbergi* from 14 to 85 days. It also reacted with the serum from *T. luwenshuni* infected sheep. However, there was no cross-reactivity with sera from animals infected with *Babesia motasi* and *Anaplasma ovis*. Comparison of the ICS with the rTuIP antigen based indirect enzyme-linked immunosorbent assays (ELISA) using test field samples showed good correlations with 93.1 % (81/87) sensitivity and 100 % (40/40) specificity, respectively, with an almost perfect agreement (Kappa = 0.895, *P* < 0.01).

**Conclusion:**

An immunochromatographic strip test based on a recombinant *T. uilenbergi* immunodominant protein (rTuIP) was developed. This is a rapid test (approximately 15 min to completion) for the detection of *T. uilenbergi* and/or *T. luwenshuni* infection that is easy to perform and; delivers results that are visible to the naked eye.

## Background

*Theileria uilenbergi* and *T. luwenshuni* are tick-borne protozoan parasites. They are causative pathogens of theileriosis of small ruminants in China [1]. The known transmission vectors for both parasites are *H. qinghaiensis* and *H. longicornis* [2–4]. Occurrence of theileriosis has been reported in many regions in China, including Gansu, Qinghai, Sichuan, Liaoning, Shanxi, Inner Mongolia, Ningxia, Xinjiang and Hubei provinces [5–9]. The disease mainly causes fever, anemia, icterus and can be fatal in goats and sheep. As such, it has restricted the development and productivity of the small ruminant livestock industry in the endemic regions [7].

The most practical and commonly used method for diagnosis of theileriosis by the veterinarian is examination of piroplasm of *T. uilenbergi* and/or *T. luwenshuni* in Giemsa-stained blood smears using a light microscope [10]. This method is reliable for the detection of infection in acute cases, but it is limited for the diagnosis of chronic cases because of the low number of parasitemia in ruminants [11]. Additionally, it is impossible to distinguish *T. uilenbergi* from *T. luwenshuni* due to their similar morphological shape. In recent years, nuclear acid based diagnostic methods have been developed to efficiently detect and/or differentiate the 2 parasites, such as polymerase chain reaction (PCR) [12–15], reverse line blot (RLB) hybridization assays [16, 17], multiplex PCR (mPCR) [18] and loop-mediated isothermal amplification (LAMP) [19]. Although these provide reliable and unambiguous pathogen detection methods for the diagnosis of the infection, they are impractical for field diagnosis since equipped laboratories are required. Regarding serological tests, several ELISAs have been established*.* The ELISA based on crude antigen (merozoite lysate) can detect antibodies against both *T. uilenbergi* and *T. luwenshuni* [20]; however preparation and standardization of the crude antigen is difficult. Alternatively, an ELISA based on the recombinant protein rTuIP has been developed and validated, which is suitable for epidemiological studies and large-scale studies [21, 22]. However, the rTuIP based ELISA is still limited as it is a laboratory test that requires professional personnel, special laboratory materials and equipment. Hence, a convenient and rapid test, such as an immunochromatographic strip is needed for the use in both clinical and field applications for the diagnosis of ovine theileriosis in China.

The colloidal gold-based immunochromatographic strip is easy to perform in the field and does not require expensive instruments. It has been widely used for the detection of infection with pathogens such as *T. annulata*, canine parvovirus, *Leptospira*, *Trichinella* in the veterinary field [23–26]. The aim of the present study was to develop a simple, portable and rapid immunochromatographic strip for the detection of *T. uilenbergi* and/or *T. luwenshuni* infections in sheep and goats.

## Methods

### Source of serum samples

Serum samples were prepared as described previously [21]. *Theileria*-free sheep were examined for merozoites in Giemsa-stained blood smears under the microscope and by PCR detection. Negative serum samples were collected before experimental infection. Sheep (No. 2203, 1236, 1219) were inoculated using blood-infected with *T. uilenbergi*; sheep (No. 1250, 1240, 1229) were attached with ticks collected in a *Theileria*-endemic region (Lintan). Serum samples were taken at 14, 19, 52 and 85 days, post infection.

One hundred and twenty seven serum samples were collected at random from Lintan County, in Gansu Province, which had been tested previously by using both the merozoite antigen ELISA and rTuIP ELISA [20, 21]. Positive sera of *T. luwenshuni*, *B. motasi* and *A. ovis* were prepared from *T. luwenshuni*, *B. motasi* and *A. ovis*-infected sheep, respectively, as described previously [21].

### Ethical approval

This study was approved by the Animal Ethics Committee of the Lanzhou Veterinary Research Institute. Care and maintenance of animals was in accordance with institutional guidelines of the Lanzhou Veterinary Research Institute, Chinese Academy of Agricultural Sciences.

### Preparation of antigen and polyclonal antibodies

Recombinant TuIP was prepared as described by Liu et al*.* [21]. Briefly, N-terminal region of the TuIP gene was cloned into the pQE31 expression vector, generating in a recombinant protein of 382 amino acids with a predicted molecular mass of 41.7 kDa. The protein product was termed rTuIP as previous reported [21]. Subsequently, the rTuIP protein was purified according to the QiaExpressionist protocols (Qiagen, Hilden, Germany). Rabbit anti-rTuIP polyclonal antibody was prepared as reported in a previous study [21].

### Preparation of the ICS

Colloidal gold was prepared according to the published method [27]. Briefly, 2 mL of 1 % trisodium citrate (w/v) was added quickly to 100 mL of 0.01 % HAuCl4 solution (w/v) heated to 90 °C and boiled for 15 min under constant stirring. As the solution cooled to room temperature (RT), the pH was adjusted to 9.0 using 0.01 M potassium carbonate and sodium azide added to a final concentration of 0.02 % (w/v). Two hundred microliter of rTuIP (2 mg/ml) was added to 20 ml colloidal gold solution and stirred 15 min followed by 4 ml 5 % BSA to block non-specific binding sites. The resulting colloidal gold-rTuIP was centrifugated at 10,000 rpm 40 min and the resuspended pellet in 1.2 ml of resuspension buffer was sprayed onto glass fiber pads and, then dried at 37 °C 30 min. Nitrocellulose membrane was incubated with rTuIP at the T line and an anti-rTuIP antibody at the C line and then dried at 37 °C for 30 min. The sample pad, conjugate pad, nitrocellulose membrane and absorbent pad were assembled on a backing plate in the appropriate order [26]. The finished plate was cut into 2.5 mm × 80 mm strips using a cutting machine. In a test, the sample was added to the sample pad where the liquid migrated towards the conjugated pad. The antibody was captured by the colloidal gold-rTuIP on the conjugated pad and resulting complex migrated to the next section of the strip which was the reaction matrix. If the sample contained anti-*Theileria* antibodies, the complex would react with the immobilized rTuIP antigen on the “T” line and a positive signal would be indicated with a red band. If the sample did not contain anti-*Theileria* antibodies, the free conjugate would migrate along the membrane towards the “C” line where it would interact with immobilized anti-rTuIP antibodies. The rest of the solution is trapped in the absorbent pad. If both the “T” line and the “C” line formed color bands, the sample was considered positive. If only the “C” line but not the “T” line formed color band, the sample was considered to be negative. The absence of a “C” line would indicate test failure.

### Preliminary evaluation of the ICS

To test specificity of the ICS, positive sera from *T. luwenshuni*, *B. motasi* and *A. oivs* and negative sera from healthy sheep were tested. The sensitivity of the ICS was evaluated with positive sera which was diluted at 1:50, 1:100, 1:200, 1:250, 1:300, and 1:500.

Field samples (*n* = 127) were tested with the ICS and compared to previously generated data using rTuIP based ELISA [21]. The correlation of the two methods was evaluated in terms of the sensitivity and specificity using the following formulas: sensitivity (Se = (No. of samples positive in both tests/total number of positive samples in the reference test) × 100), specificity (Sp = (No. of samples negative in both tests/total number of negative samples in the reference test) × 100). The Kappa test was used to calculate the degree of agreement between the ICS and rTuIP ELISA using SPSS 18.0.0 software for Windows (SPSS Inc., Chicago, IL, USA) [28].

## Results

### Establishment of the ICS

A range of concentration of colloidal gold-rTuIP on the conjugated pad, rTuIP on the “T” line and rabbit anti-rTuIP polyclonal antibody on the “C” line were used to optimize the ICS system. The optimal concentration of the colloidal gold-rTuIP on glass fiber pads and the rTuIP on the “T” line were 0.02 mg/ml and 0.5 mg/ml, respectively. A dilution of 1:15 was found to be optimal for the rabbit anti-rTuIP polyclonal antibody on the “C” line for the subsequent experiment. For testing serum samples, 50 to 100 μl samples were loaded onto the sample pad, and the result was visually detected to the naked eye within 15 min.

### Evaluation of the ICS

Analysis of serum samples collected at days, 0, 14, 19, 52 and 85 from the experimentally infected animals was done using the ICS. The results demonstrated that the ICS was able to detect antibodies from days 14 to 85 (Table 1), indicating that the test is suitable for the detection of *T. uilenbergi* infection.

**Table 1:** Detection of anti-TuIP specific antibody in sera from sheep experimentally infected with *T. uilenbergi* taken at daily intervals post-infection using the immunochromatographic strip

	Experimental infected sheep					
	Infectious blood inoculation			Infected *H. qinghaiensis* infestation		
	2203	1236	1219	1250	1240	1229
0 days p.i.	+/−	-	-	-	-	-
14 days p.i.	+	+	+	+	+	+
19 days p.i.	+	+	+	+	+	+
52 days p.i.	+	+	+	+	+	+
85 days p.i.	+	+	+	+	+	+

“+” indicates a positive result, “-” a negative result, “+/−” a suspected result, p.i. post infection, Sheep Nos. 1250, 1240 and 1229 were infected by feeding 200 adult *Heamaphysalis qinghaiensis* ticks collected from Lintan, China, Sheep Nos. 2203, 123681 and 1219 were infected by blood inoculation with *T. uilenbergi* Lintan isolate

**Fig. 1:**
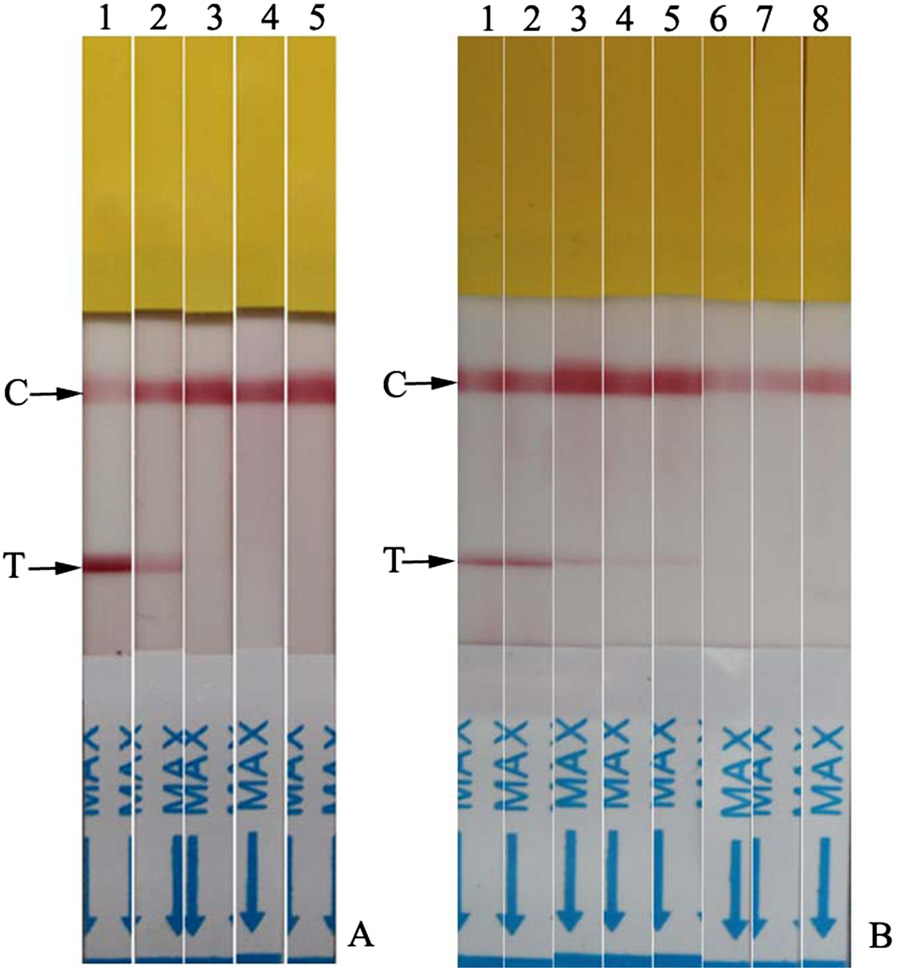
**a**, Testing for cross-reactivity using the immunochromatographic strip. Serum samples were from sheep infected with *Theileria uilenbergi*, *T. luwenshuni*, *Anaplasma ovis* and *Babesia motasi*. Serum from an uninfected sheep was used as negative control. Lane 1: positive serum of *T. uilenbergi*, Lane 2: positive serum of *T. luwenshuni*, Lane 3: positive serum of *A. ovis*, Lane 4: positive serum of *B. motasi*, Lane 5: negative control. C control line, T test line. **b**, The sensitivity of the immunochromatographic strip. Lane 1 to lane 6 positive serum from a *T. uilenbergi*-infected sheep diluted at 1:50, 1:100, 1:200, 1:250, 1:300, 1:500. Lane 7: dilution buffer used as blank control, Lane 8: serum from an uninfected sheep used as negative control

When sera from sheep experimentally infected with *T. luwenshuni*, *B.motasi* and *A. ovis* were tested with the ICS, a positive cross-reaction was observed from serum from the closely related pathogen, *T. luwenshuni*. No cross-reaction was observed from negative serum or positive sera of sheep experimentally infected with *B. motasi* and *A. ovis* (Fig. 1a). To assess analytical sensitivity, positive serum samples diluted at 1:50, 1:100, 1:200, 1:250, 1:300, and 1:500 were tested with the ICS. The red line could be observed clearly at both the T and C lines when the dilution of the serum samples was no less than 1:200. A weak red line at the T line could still be detected at 1:250 dilution. This result suggested that the ICS could detect antibodies in serum samples at very low concentrations (Fig. 1b).

In a preliminary run, the ICS was evaluated for its reliability by testing anti-TuIP-specific antibodies against 127 field samples under optimized conditions, and comparing the results obtained with serum samples using TuIP-ELISA [21]. When compared to the TuIP-ELISA as reference test, the correlation in terms of the sensitivity and specificity was 93.10 % (81/87) and 100 % (40/40), respectively, with an almost perfect agreement (Kappa 0.895, *P* < 0.01) (Table 2).

**Table 2:** Comparison of the immunochomatographic strip with the rTuIP-ELISA as the reference test for detection of antibodies in 127 field samples from sheep

		rTuIP-ELISA		
		Positive	Negative	Total
ICS	Positive	81	0	81
	Negative	6	40	46
	Total	87	40	127

Sensitivity of the ICS = 93.1 %; specificity of the ICS = 100 %; measure of agreement of the two tests, Kappa = 0.895, *P* < 0.01

## Discussion

The ICS is a portable and rapid method for the detection of antibodies in serum and has been widely applied for the diagnosis of protozoa parasite infections, such as *Babesia* [29–31], *Toxoplasma* [32, 33]. The merits of ICS include; increased speed, it is simple to use, and it is easy-to-conduct on site with no requirement of laboratory instruments. Moreover, the result is visible and easily detected by the naked eye. Despite these advantages, the ICS has not yet been developed for the detection of *T. uilenbergi* and *T. luwenshuni* infection.

TuIP is an antigen of *T. uilenbergi*, and the recombinant TuIP has been successfully used for the development of an indirect ELISA for the detection of *T. uilenbergi* and *T. luwenshuni* infection [21, 22]. The rTuIP was therefore chosen as an antigen to develop the ICS in this study. Besides the antigen, several critical aspects in the establishment of an ICS had to be considered, such as the quality and concentration of antigen, antibody, and/or colloidal gold particles, and the buffer system [23, 34]. To optimize the ICS, different concentration of the colloidal gold-rTuIP on the conjugated pad, rTuIP antigen on the “T” line and rabbit anti-rTuIP polyclonal antibody on the “C” line were tested.

When testing for potential cross-reactivity of the ICS using *T. luwenshuni*, *B. motasi* and *A. ovis*, the results showed that the ICS could also detect *T. luwenshuni* infection, but not *Babesia* and *Anaplasma* infections. This is accordance with the results obtained from the rTuIP based ELISA in our previous studies [21, 22]. It is known that *T. uilenbergi* and *T. luwenshuni* are very closely related pathogens in terms of morphology, transmission vectors, hosts, and distribution in the endemic regions as well as phylogenetic relationship [35, 36]. Therefore it was assumed that TuIP of *T. uilenbergi* might share similar epitopes in *T. luwenshuni* [22].

Testing of the serum samples from experimentally infected sheep by time course at days 14,19,52,85 gave positive results that correlated to the rTuIP based ELISA [21]. These results indicate that the ICS is suitable for the detection of the infection caused by the *Theileria* parasites both during the early stage and the chronic phase. Further evaluation of ICS was done by testing 127 field serum samples and comparing the results with data obtained by investigation of serum samples using the rTuIP based ELISA. The comparison showed that the ICS had an almost perfect agreement to the rTuIP based ELISA (Kappa = 0.895, *P* < 0.01) in terms of the sensitivity of 93.1 % and the specificity of 100 %, indicating that the ICS would be reliable for use in the field.

## Conclusion

An immunochromatographic strip based on a recombinant *T. uilenbergi* immunodominant protein (rTuIP) was developed for the detection of *T. uilenbergi* and/or *T. luwenshuni* infection in the field. The sensitivity and specificity were in good agreement with the rTuIP-based indirect ELISA [21]. This method is not able to differentiate *T. uilenbergi* infection from *T. luwenshuni* infection, however it is easily applicable by veterinarians for the on-site diagnosis of ovine theileriosis.
